# Symptoms of periodontitis and antibody responses to *Porphyromonas gingivalis* in juvenile idiopathic arthritis

**DOI:** 10.1186/s12969-016-0068-6

**Published:** 2016-02-09

**Authors:** Lauren Lange, Geoffrey M. Thiele, Courtney McCracken, Gabriel Wang, Lori A. Ponder, Sheila T. Angeles-Han, Kelly A. Rouster-Stevens, Aimee O. Hersh, Larry B. Vogler, John F. Bohnsack, Shelly Abramowicz, Ted R. Mikuls, Sampath Prahalad

**Affiliations:** Ann and Robert H. Lurie Children’s Hospital of Chicago, Chicago, IL USA; VA Nebraska-Western Iowa Health Care System and University of Nebraska Medical Center, Omaha, NE USA; Department of Pediatrics Emory University School of Medicine, 1760 Haygood Dr. NE, Atlanta, GA 30322 USA; Children’s Healthcare of Atlanta, Atlanta, GA USA; University of Utah School of Medicine, Salt Lake City, UT USA; Department of Surgery, Division of Oral and Maxillofacial Surgery, Emory University School of Medicine, Atlanta, GA USA; Department of Human Genetics, Emory University School of Medicine, Atlanta, GA USA

**Keywords:** Juvenile idiopathic arthritis, Anti-citrullinated peptide antibody, Periodontitis, *Porphyromonas gingivalis*, *Prevotella intermedia*

## Abstract

**Background:**

The association between rheumatoid arthritis (RA) and periodontitis is well established. Some children with juvenile idiopathic arthritis (JIA) phenotypically resemble adults with RA, characterized by the presence of anti-cyclic citrullinated peptide (CCP) antibodies. We sought to investigate an association between CCP-positive JIA and symptoms of periodontitis and antibodies to oral microbiota.

**Methods:**

Antibodies to oral pathogens *Porphyromonas gingivalis*, *Prevotella intermedia,* and *Fusobacterium nucleatum* were measured using ELISA in 71 children with CCP-positive JIA and 74 children with CCP-negative JIA. Oral health history was collected from 37 children with CCP-positive JIA and 121 children with CCP-negative JIA. T-tests, Chi-square tests, Mann–Whitney U tests, and multivariable regression were used to compare the groups.

**Results:**

Compared to those with CCP-negative JIA, children with CCP-positive JIA were more likely to be female, older and non-Caucasian. Anti-*P. gingivalis* (*p* <0.003) and anti-*P. intermedia* (*p* <0.008) IgG antibody titers were higher in the CCP-positive cohort. Differences in *P. gingivalis* antibody titers remained significant after adjusting for age (*p* = 0.007). Children with CCP-positive JIA more likely reported tender/bleeding gums (43 % vs. 24 %, *p* < 0.02) compared to children with CCP-negative JIA. After controlling for age at collection, the odds of having tender/bleeding gums were 2.2 times higher in the CCP-positive group compared (95 % CI 0.98 – 4.83; *p* = 0.056).

**Conclusions:**

Children with CCP-positive JIA have higher antibody titers to *P. gingivalis* and more symptoms of poor oral health, supporting a possible role for periodontitis in the etiology of CCP-positive JIA.

## Background

Rheumatoid arthritis (RA) is among the most common forms of inflammatory arthritis in adults with a majority of patients demonstrating positivity for rheumatoid factor (RF) and/or anti-cyclic citrullinated peptide (CCP) antibodies. About 5 % of children with juvenile idiopathic arthritis (JIA) have a disease that phenotypically resembles RA, characterized by chronic inflammatory arthritis and the presence of RF and/or CCP. Such children likely represent a subset of seropositive RA patients with childhood onset of RA [1]. It is not known if childhood onset RA shares the same risk factors associated with adult-onset RA.

Both genetic and environmental factors are associated with the risk of developing RA in adults. Meta-analysis of RA genome-wide studies have identified 98 candidate genes at 101 loci that are definitely associated with RA [2]. We have shown that children with RA demonstrate associations with most of the *HLA-DRB1* alleles encoding the shared epitope [3], as well as several non-HLA loci [4]. These studies suggest that childhood onset RA shares many of the genetic risk factors of its adult onset counterpart.

There is also substantial evidence to support environmental factors contributing to RA susceptibility. A significant gene-environment interaction between cigarette smoking and shared epitope-encoding *HLA-DR* alleles in subjects with CCP-positive RA was demonstrated by Klareskog et al. [5]. An association between periodontitis and RA has also been established [6]. A prevailing hypothesis suggests that in genetically susceptible individuals citrullinated peptides produced by *Porphyromonas gingivalis* may disrupt the immune system’s acceptance of endogenous citrullinated antigens, leading to a robust immune response to both self- and non-self citrullinated antigens. *P. gingivalis* is a Gram-negative anaerobe implicated in periodontitis pathogenesis and is the only prokaryote known to have the capacity to citrullinate exogenous antigen.

Association studies of JIA and oral health compared oral health in children with JIA to controls with conflicting results [7–9]. To our knowledge, there have been no published investigations of antibody response to *P. gingivalis* or other oral pathogens and CCP-positive JIA. We evaluated the hypothesis that the prevalence of anti-*P. gingivalis* antibodies, and poor oral hygiene would be higher among children with CCP-positive JIA, compared to children with JIA negative for CCP antibodies.

## Methods

### Study population

**Fig. 1:**
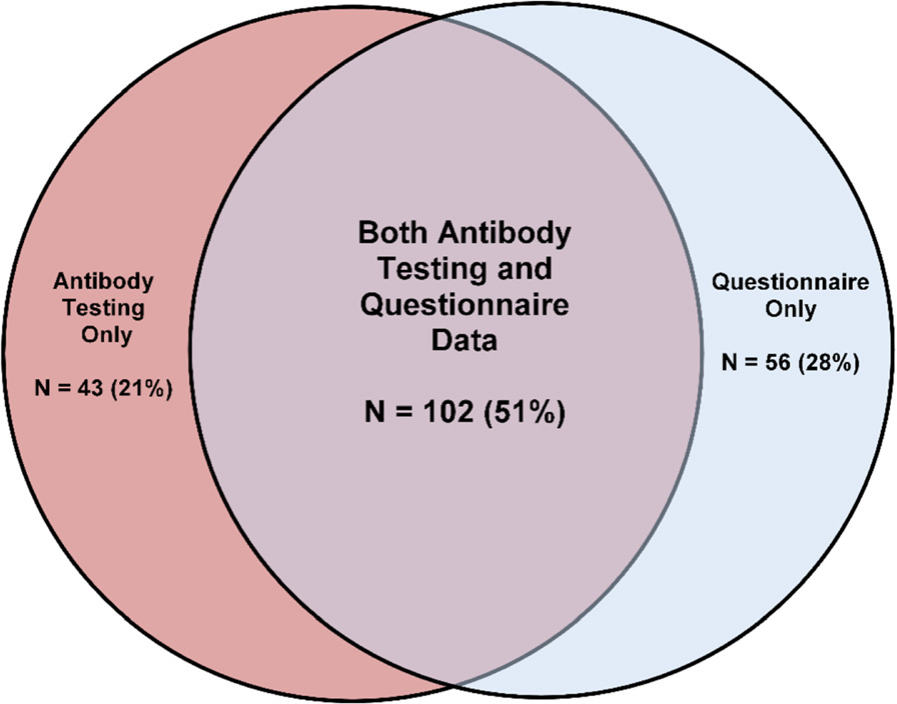
Venn diagram that depicts the number of subjects that completed the oral health questionnaire, who underwent antibody testing for oral pathogens or both

Children satisfying International League of Associations for Rheumatology (ILAR) classification criteria for JIA [10] were enrolled from pediatric rheumatology clinics at the University of Utah and Emory University School of Medicine under protocols approved by the respective institutional review boards. Individuals comprising the CCP-positive cohort included children diagnosed with JIA and at least one documented positive anti-CCP antibody titer, with or without a positive test for RF. Individuals comprising the CCP-negative cohort included children with JIA and a documented negative anti-CCP antibody test, as well as a negative RF. Not all subjects used for the serological studies completed the questionnaire which was administered in clinic (Fig. 1).

### Oral health and tobacco smoke exposure questionnaires

Oral health and tobacco smoke exposure questionnaires were administered to the parents of children with JIA who presented to the pediatric rheumatology clinics at Emory University School of Medicine between April and August 2013. The questionnaire included questions regarding frequency of dental check-ups, number of cavities, frequency of teeth brushing and flossing, and the presence of several symptoms of periodontitis including persistent bad breath, recession of gums, red/swollen gums, tender/bleeding gums, loose permanent teeth, and pain on chewing.

### Antibody testing

Serum was separated soon after blood draw and stored at −80 °C until investigated. Assays were performed in a single batch by laboratory personnel blinded to autoantibody status or other clinical data. IgG antibodies to the outer membrane antigens of *P. gingivalis*, *Prevotella intermedia,* and *Fusobacterium nucleatum* were measured using ELISA protocol in both the CCP-positive and CCP-negative cohorts as previously described [11].

### Data analysis

Statistical analyses were performed using SAS 9.3; statistical significance was assessed at *p* < 0.05. Descriptive statistics were calculated for all variables of interest and include means and standard deviations, median and ranges, or counts and percentages, when appropriate. Patient and clinical characteristics were compared between patient subgroups using T-tests and Mann Whitney-U tests for continuous data and Chi-square tests for categorical data. Normality of antibody concentrations was assessed using histograms, density plots, and the Anderson-Darling test for normality. If normality was suspect, the natural log transformation was applied and normality was reassessed. If the transformation failed to normalize the data, the analysis was carried out non-parametrically by using the ranked data. To examine the potential impact of race/ethnicity on these comparisons, pre-planned sub-analyses were completed among non-Hispanic Caucasians who comprised the largest patient group. Multivariable linear regression was used to examine the effect of CCP-positivity on antibody levels while adjusting for age. A logistic regression was also performed to compare signs and symptoms of periodontal disease among CCP groups (positive vs. negative) while adjusting for patient age. Spearman’s rank-order correlation coefficient with associated 95 % confidence intervals was used to assess the relationship between different antibody titers and among patients with and without periodontal disease. For this purpose, periodontal disease was defined as having any of the following oral health problems: tender or bleeding gums, red or swollen gums, loose teeth.

## Results

### Patient characteristics

Seventy-seven children with CCP-positive JIA and 124 children with CCP-negative JIA were included. Of the CCP-positive group, 65 children were both CCP-positive and RF-positive, whereas 12 children were CCP-positive, but RF-negative. All 124 children with CCP-negative JIA were negative for both CCP and RF. Children with CCP-positive JIA were significantly more likely to be female (89.6 % vs. 65.2 %, *p* < 0.001), older at diagnosis (10.4 vs. 6.0 years, *p* < 0.001), and non-Caucasian (42.9 % vs 6.3 %, *p* <0.001) compared to the CCP negative group (Table 1).

**Table 1:** Clinical and demographic characteristics of study participants

	Group		*P*-value
	Anti-CCP (+) JIA (*N* = 77)	Anti-CCP (−) JIA (*N* = 124)	
Age at Diagnosis, years, *Mean ± SD*	10.4 ± 4.3	6.0 ± 4.4	**<0.001**
Age at Collection, years, *Mean ± SD*	13.3 ± 3.8	9.7 ± 4.4	**<0.001**
Gender- Female, N (%)	69 (89.6 %)	81 (65.3 %)	**<0.001**
Race, *N* (%)			
Caucasian	44 (57.1 %)	105 (84.7 %)	**<0.001**
African American	23 (29.9 %)	12 (9.7 %)	
Other^a^	10 (13.0 %)	7 (5.6 %)	
Hispanic, *N* (%)	13 (16.9 %)	11 (8.9 %)	0.089
ILAR, N (%) (*n* = 199)			
Systemic	0 (0.0 %)	13 (10.6 %)	**0.003**
RF (+)	59 (76.6 %)	0 (0.0 %)	**<0.001**
RF (−)	5 (6.5 %)	46 (37.7 %)	**<0.001**
Oligo Persistent	4 (5.2 %)	30 (24.6 %)	**<0.001**
Oligo Extended	4 (5.2 %)	12 (9.8 %)	0.386
ERA	1 (1.3 %)	20 (16.4 %)	**<0.001**
Psoriatic	1 (1.3 %)	0 (0.0 %)	0.766
Other	3 (3.9 %)	1 (0.8 %)	0.178

*CCP* cyclic citrullinated peptide, *RF* rheumatoid factor, *ERA* enthesitis related arthritis, JIA was classified according to International League of Associations for Rheumatology Criteria. Children with CCP-positive JIA had at least one documented positive titer for anti-CCP antibodies, with or without a positive test for RF. Children with CCP-negative JIA tested negative for both anti-CCP antibodies and RF. Other race defined as Asian, Bi-racial, or other race not denoted by one of the categories

### Antibody testing

**Fig. 2:**
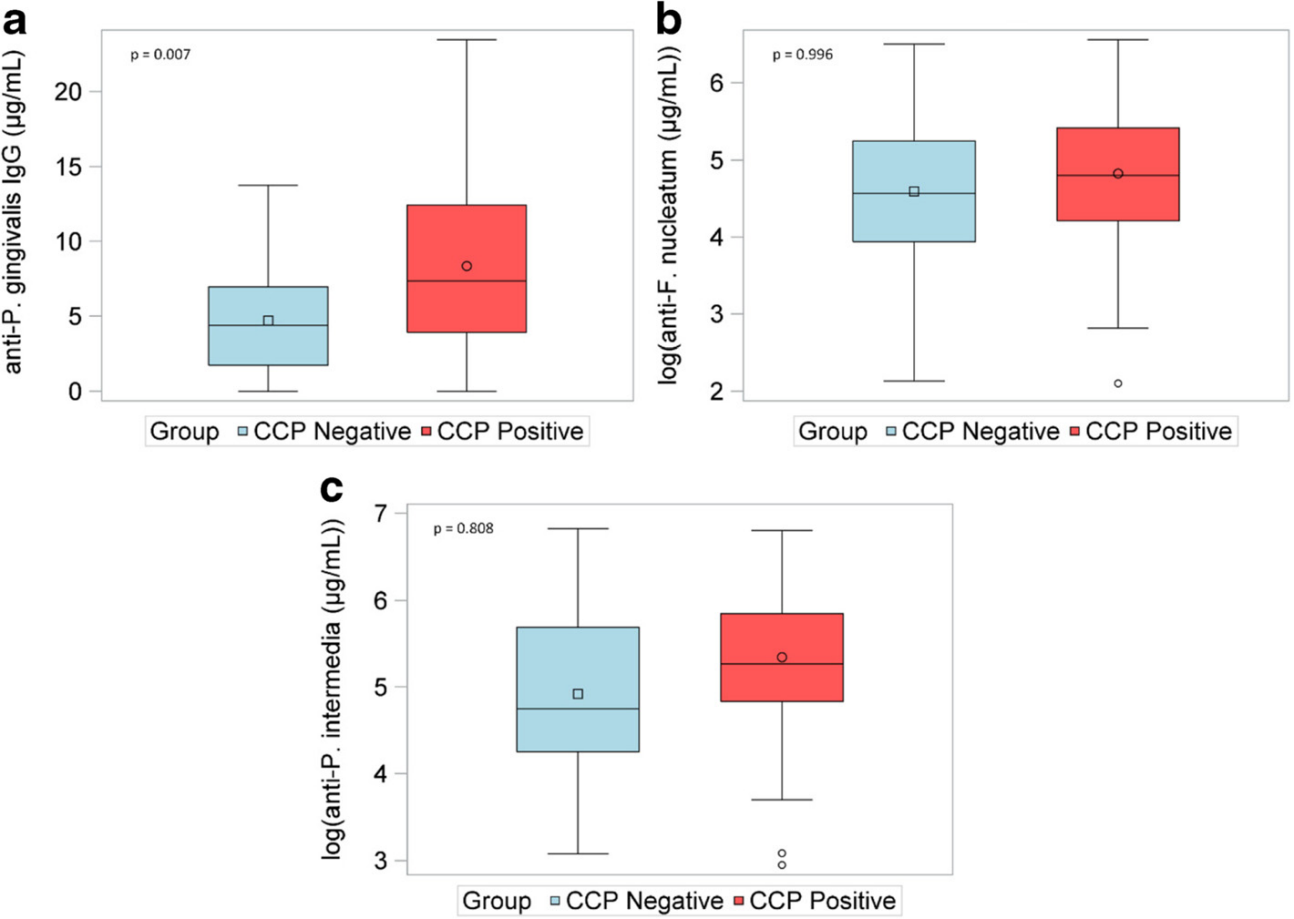
Boxplots represent age-adjusted concentrations of antibody to *Porphyromonas gingivalis*, and log transformed concentrations of antibody to *Fusobacterium nucleatum*, and *Prevotella intermedia* in 74 children with CCP-negative (*blue*) and 71 children with CCP-positive JIA (*red*). Although all subjects were included in the non-parametric analysis of *P gingivalis* antibody concentrations, 3 outliers with CCP-negative and 2 outliers with CCP-positive JIA were removed from Fig. 1a for illustration purposes

Serum anti-bacterial antibodies were measured in 71 children with CCP-positive JIA and 74 children with CCP-negative JIA. Because these antibody titers were not normally distributed, the data were log transformed, normalizing the distribution for antibody to *P. intermedia* and *F. nucleatum*. However, the distribution of anti- *P. gingivalis* antibodies remained left-skewed despite log transformation; therefore, nonparametric analyses were used. Anti-*P. gingivalis* antibody concentrations were higher in the CCP-positive cohort compared to children with CCP-negative JIA (median: 9.04 μg/mL vs. 5.69 μg/mL; *p* < 0.001), Fig. 2. Log concentrations of anti-*P. intermedia* antibody titers were also significantly higher in the CCP-positive cohort (5.4 ± 0.8 μg/mL vs. 4.9 ± 1.0 μg/mL; *p* < 0.005). Log concentrations of Anti-*F. nucleatum* IgG were not different between the two groups. Age at time of collection was significantly different between CCP-positive and negative JIA patients (13.3 ± 3.8 vs. 9.7 ± 4.4; *p* < 0.001). As a result, multivariable regression was used to examine the effect of CCP-positivity while adjusting for age differences between the groups. After adjusting for age, anti-*P gingivalis* concentrations remained significant (*p* = 0.007). In contrast, log concentrations of anti-*P intermedia* were no longer significantly different between the two groups (*p* = 0.808).

**Fig. 3:**
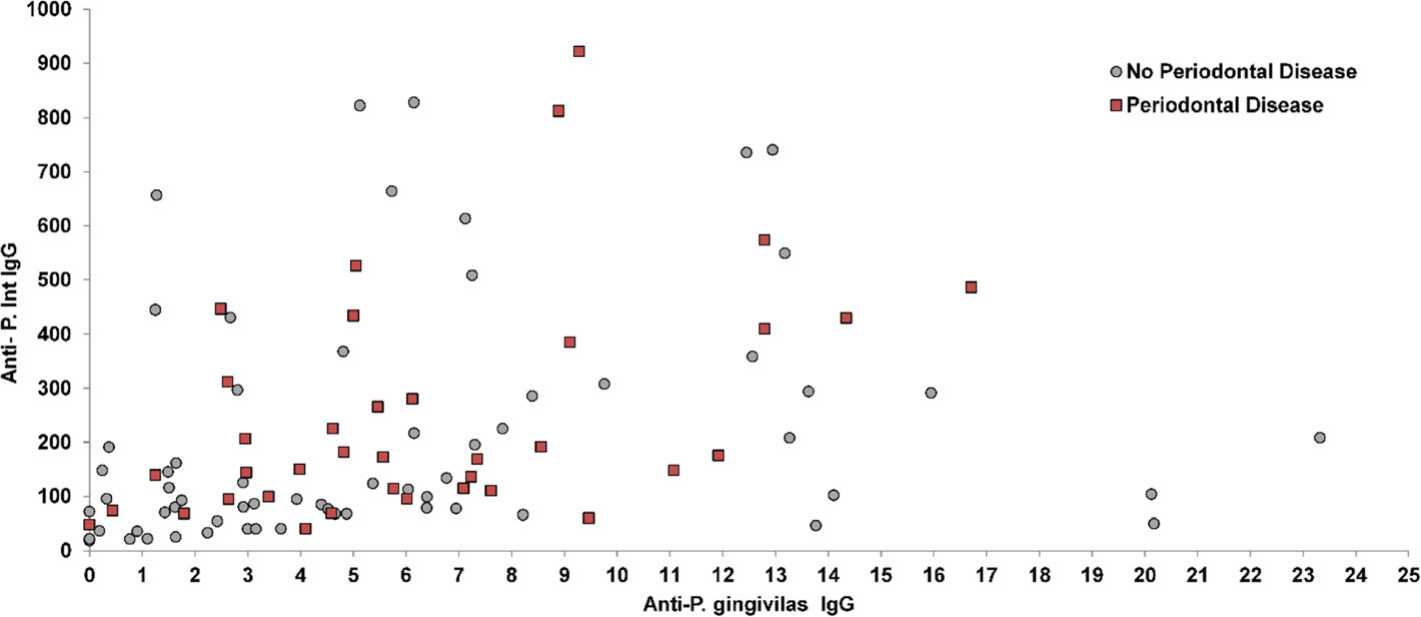
Scatterplot depicting the relationship between *P. gingivalis* and *P. intermedia* in subjects with and without periodontal disease revealed similar relationships between the two antibodies with correlations of 0.43 and 0.44 respectively. There did not appear to be synergistic effect between the two antibodies

There was a moderate strong positive relationship between *P. gingivalis* and *Prevotella intermedia* (r_s_ = 0.50; 95 % CI (0.37 – 0.61); *p* < 0.001) and with *Prevotella intermedia,* and *Fusobacterium nucleatum* (r_s_ = 0.45; 95 % CI (0.31 – 0.58); *p* < 0.001). In contrast, there was a weak association between *P. gingivalis* and *Fusobacterium nucleatum* (r_s_ = 0.20; 95 % CI (0.04 – 0.35); *p* = 0.016). Further examination of the relationship between *P. gingivalis* and *P. intermedia* in patients with and without periodontal disease (Fig. 3), revealed similar relationships between the two antibodies with correlations of 0.43 and 0.44, respectively. Furthermore, there did not appear to be a synergistic effect between the two antibodies.

### Oral health history

The oral health and smoke exposure questionnaire was completed by 37 individuals with CCP-positive JIA and 121 with CCP-negative JIA. The CCP-positive JIA cohort had an increased prevalence of tender or bleeding gums compared to the CCP-negative JIA cohort (43 % vs 23 %, *p* = 0.017) (Table 2). Children with CCP-positive JIA also had a higher prevalence of red/swollen gums compared to children with CCP-negative JIA (35 % vs. 20 %, *p* = 0.055). When the oral health comparisons were restricted to non-Hispanic white subjects, red/swollen gums (55 % vs 19 %. *p* <0.003) and tender bleeding gums (60 % vs 21 %, *p* <0.002) were significantly more prevalent among children with CCP-positive JIA, compared to children with CCP-negative JIA. In order to investigate if current or past temporomandibular joint (TMJ) arthritis contributed to poor oral hygiene, we analyzed 84 subjects on whom we had detailed TMJ involvement history (Table 3). There was no association between TMJ involvement and tender/bleeding gums. Children with TMJ involvement had a higher prevalence of loose teeth which was not statistically significant.

**Table 2:** Self-reported dental hygiene characteristics and symptoms of periodontitis among study participants

Characteristic	Group		*P*-value
	Anti-CCP (+) (*N* = 37)	Anti-CCP(−) (*N* = 121)	
Signs and Symptoms of periodontis^1^, *N* (%)			
Tender Bleeding Gums	16 (43.2 %)	28 (23.1 %)	**0.017**
Red Swollen Gums	13 (35.1 %)	24 (19.8 %)	0.055
Pain on chewing	9 (24.3 %)	16 (13.2 %)	0.105
Bad Breath	7 (18.9 %)	28 (23.1 %)	0.588
Gums Pulled away from Teeth	3 (8.1 %)	8 (6.6 %)	1.000
Loose Teeth	1 (2.7 %)	5 (4.1 %)	1.000
Dental Hygiene ^1^			
≥2 Check-ups per year, *N* (%)	28 (75.7 %)	87 (72.5 %)	0.703
Brushing at least 2x per day, *N* (%)	24 (64.9 %)	77 (63.6 %)	0.892
Flossing at least 1 per day, *N* (%)	9 (26.5 %)	41 (36.9 %)	0.261
Cavities, *N* (%)	24 (64.9 %)	81 (66.9 %)	0.815

^1^ *CCP* cyclic citrullinated peptide

Oral health questionnaire completed by parents of children with CCP-positive JIA seen in clinic during the study period. Statistically significant *p* value bolded

**Table 3:** Self-reported dental hygiene characteristics and symptoms of periodontitis among study participants with or without TMJ involvement

Characteristic	Group		*P*-value
	TMJ Involvement (*N* = 23)	No TMJ Involvement (*N* =61)	
Signs and Symptoms of periodontis^1^, *N* (%)			
Tender Bleeding Gums	5 (21.7 %)	13 (21.3 %)	1.00
Red Swollen Gums	4 (17.4 %)	15 (24.6 %)	0.482
Pain on chewing	5 (21.7 %)	6 (9.8 %)	0.275
Bad Breath	7 (30.4 %)	15 (24.6 %)	0.589
Gums Pulled away from Teeth	2 (8.7 %)	3 (4.9 %)	0.611
Loose Teeth	3 (13.0 %)	1 (1.6 %)	0.061
Dental Hygiene ^1^			
≥2 Check-ups per year, *N* (%)	18 (78.3 %)	46 (75.4 %)	0.784
Brushing at least 2x per day, *N* (%)	16 (69.6 %)	31 (50.8 %)	0.123
Flossing at least 1 per day, *N* (%)	7 (33.3 %)	15 (27.3 %)	0.602
Cavities, *N* (%)	14 (60.8 %)	40 (65.6 %)	0.688

^1^ *CCP* cyclic citrullinated peptide, *TMJ* temporomandibular joint

Oral health questionnaire completed by parents of children with CCP-positive JIA seen in clinic during the study period. Jaw involvement included current or prior history of temporomandibular joint arthritis

## Discussion

Studies in adults with RA have revealed an association between seropositive RA and periodontitis [12]. Furthermore, elevated antibody responses to *P. gingivalis* have been demonstrated in adults with CCP-positive RA [13, 14]. To our knowledge, ours is the first investigation into antibody responses to oral pathogens and clinical features of periodontitis of children with CCP-positive JIA. We found that children with CCP-positive JIA have increased antibody responses to *P. gingivalis* and *P. intermedia*, but not *F. nucleatum* compared to children with CCP-negative JIA. Our results support the hypothesis that *P. gingivalis* might play a role in the pathogenesis of CCP-positive JIA similar to RA. We also discovered elevated antibody responses to *P. intermedia* in children with CCP-positive JIA. *P. intermedia* is also known to cause periodontitis, and may also be associated with RA [15]. Furthermore, the presence of both *P gingivalis* and *P intermedia* may act synergistically, increasing periodontitis risk by almost 6-fold [16].

We also demonstrated a statistically significant increase in the prevalence of bleeding/tender gums in children with CCP-positive JIA. Increased prevalence of red/swollen gums was also observed in the CCP-positive JIA cohort, but this result was not statistically significant, perhaps reflecting our limited sample size. To address the possible influence of race and ethnicity on the prevalence of symptoms of poor oral health, we restricted the analysis to non-Hispanic White subjects, which confirmed the increased prevalence of red/swollen and tender/bleeding gums in children with CCP-positive JIA. These observations support the conclusion that subclinical periodontitis may be involved in the pathogenesis of CCP-positive JIA.

Prior investigations of the association between oral health and JIA have yielded conflicting results. Miranda et al., investigated 32 JIA cases and 24 healthy controls and concluded that JIA cases demonstrate more periodontal attachment loss than controls, in spite of similar plaque and marginal bleeding levels [7]. Another investigation of 41 JIA cases and 41 controls demonstrated that frequencies of sites with plaque (32 % vs. 19 %, *P* = 0.013), bleeding on probing (26 % vs. 14 %, *P* < 0.01), and probing depth 2 mm (32 % vs. 2 %, *P* < 0.001) were higher among JIA patients [9]. By contrast, an investigation of 78 JIA cases and 75 healthy controls concluded that JIA was not a risk factor for periodontitis after adjustment for microbial plaque [8], the latter serving as a surrogate for oral hygiene status. Unlike our study, these studies did not differentiate between CCP-positive and CCP-negative JIA.

Our study has limitations. Evaluation of the gingival health of the participants through full mouth periodontal exams would have been ideal, but was beyond the scope of our study. However, we did consult pediatric dentists in the drafting of the questionnaire to evaluate oral health. Although administration of oral health questionnaires to all CCP-positive JIA cases tested for antibody responses would have been ideal, given the rarity of CCP-positive JIA, we used stored serum samples from some children with CCP-positive JIA to increase power. We did not include healthy controls. Rather our focus was to investigate the differences in oral health variables and oral bacterial antibody concentrations between children with CCP-positive JIA and children with CCP-negative JIA. Hence, the lack of healthy controls do not affect our conclusions. Also it is possible that some of the observed differences might be due to differences in ages of the subjects, but the association between CCP-positive JIA and antibody responses to *P. gingivalis* persisted despite correcting for age at collection of sample. After controlling for age the odds of having tender/bleeding gums was higher in the CCP-positive group although this was only marginally significant (*p* = 0.056) reflecting the small cohort for this analysis. An investigation in adults has shown that the prevalence of oral health problems among adults in the US varied by race/ethnicity, although socio-economic factors such as nutrition, access to care and education are major confounders [17]. In order to address the possibility the oral health symptoms in our cohort were also influenced by race, we restricted the comparison to non-Hispanic White subjects, and both red/swollen gums and tender bleeding gums were significantly more prevalent among children with CCP-positive JIA. Like most association studies, our results point to an association between poor oral health and CCP-positive JIA but do not establish causality. It is possible that arthritis related factors lead to poor oral health. We did not observe an association between temporomandibular joint involvement and poor oral health in a subset of our cohort with detailed TMJ involvement history.

## Conclusions

We have shown that children with CCP-positive JIA have increased antibody responses to *P. gingivalis* compared to children with CCP-negative JIA, suggesting that periodontitis or pathogens implicated in periodontitis might be implicated in the development of CCP-positive disease. Children with CCP-positive JIA also tend more often to have symptoms of periodontitis compared to children with CCP-negative JIA. In the future, a careful oral examination by periodontists or other qualified oral health professionals, and the simultaneous ascertainment of antibody responses to oral pathogens, as well as next-generation sequencing of the subgingival microbiome on an inception cohort of children with CCP-positive JIA would allow us to better characterize the etiopathology of this disease.
